# Distribution of health care resources in Mongolia using the Gini coefficient

**DOI:** 10.1186/s12960-017-0232-1

**Published:** 2017-08-29

**Authors:** Oyunchimeg Erdenee, Sekar Ayu Paramita, Chiho Yamazaki, Hiroshi Koyama

**Affiliations:** 1 Department of Public Health, Gunma University, 3-39-22 Showa, Maebashi, 371-8511 Japan; 2 Department of Public Health, Universitas Padjadjaran, Jl. Eycman No. 38, Bandung, Indonesia

**Keywords:** Geographic distribution, Equality, Physicians, Health resources, Mongolia

## Abstract

**Background:**

Attaining the perfect balance of health care resources is probably impracticable; however, it is possible to achieve improvements in the distribution of these resources. In terms of the distribution of health resources, equal access to these resources would make health services available to all people. The aim of this study was to compare the distributions of health care resources in urban, suburban, and rural areas of Mongolia.

**Methods:**

We compared urban and rural areas using the Mann–Whitney *U* test and further investigated the distribution equality of physicians, nurses, and hospital beds throughout Mongolia using the Gini coefficient—a common measure of distribution derived from the Lorenz curve. Two indicators were calculated: the distribution per 10 000 population and the distribution per 1000 km^2^ area.

**Results:**

Urban and rural areas were significantly different only in the distribution of physicians per population. However, in terms of the distribution per area, there were statistical differences in physicians, nurses, and hospital beds. We also found that distributions per population unit were equal, with Gini coefficients for physicians, nurses, and hospital beds of 0.18, 0.07, and 0.06, respectively. Distributions per area unit were highly unequal, with Gini coefficients for physicians, nurses, and hospital beds of 0.74, 0.67, and 0.69, respectively.

**Conclusions:**

Although the distributions of health care resources per population were adequate for the population size, a striking difference was found in terms of the distributions per geographical area. Because of the nomadic lifestyle of rural and remote populations in Mongolia, geographical imbalances need to be taken into consideration when formulating policy, rather than simply increasing the number of health care resources.

## Background

Human resources are the major building blocks of health systems [1, 2], and all health care is eventually delivered by and to people [3]. Thus, a clear picture of the allocation of physical and human resources (especially by area), the proportionate distribution of such resources, and timely revision enable the achievement of better health outcomes and health care accessibility for all [4].

### Overview of health resources in Mongolia

Mongolia is a land-locked country with a population of three million, 54.3% of whom reside in rural areas. On average, fewer than two people occupy each square kilometer [5]. The Human Development Index value for Mongolia was 0.73 in 2015 [5].

The Mongolian health administration is split into two main divisions: one for the capital city of Ulaanbaatar and one for the 21 provinces (*aimags*). Ulaanbaatar consists of nine districts, which are further divided into 152 sub-districts (*khoroos*). Likewise, each province consists of 3–28 sub-provinces (*soums).* The 329 sub-provinces are further divided into 1613 communes (*baghs*) [5]*.*

**Fig. 1:**
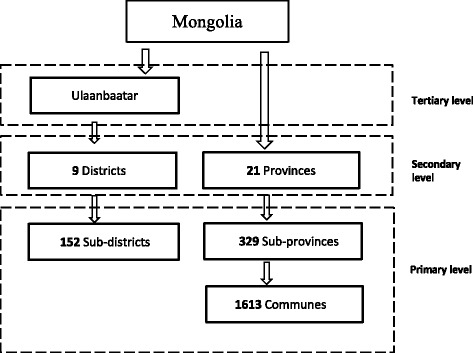
Health administrative divisions. Source: Ministry of Health [6] http://www.chd.mohs.mn/images/pdf/sma/2015/eruul_mendiin_uzuulelt_2014_angli_1.pdf. Figure 1 depicts the referring levels based on Mongolia’s administrative divisions

These administrative divisions are represented by a two-tier referral system: primary care and specialized care, which includes secondary and tertiary care (Fig. 1). Health care services are delivered by 3100 health organizations—both public and private sectors—and all specialized centers are located in Ulaanbaatar [6].

A few studies in Mongolia have focused on inequality in health. However, little attention has been paid to inequalities in health resources by geographical area [7, 8]. For this reason, the present study aimed to compare the distributions of health care resources in urban, suburban, and rural areas.

## Methods

### Setting

The data analyzed in this study were obtained from the 2014 Mongolian Health Indicators, as compiled by the Ministry of Health of Mongolia [6]. Geographical and population data were taken from the National Statistical Information Service of Mongolia [5]. All of the data used in this study were publicly available online when the study was conducted. We selected three health care resource variables for the study: numbers of physicians, nurses, and hospital beds.

We used population density to differentiate urban, suburban, and rural areas—a definition suggested by Matsumoto et al. [9]—because no standard demarcation for urban vs. rural status exists. Provinces with a population density higher than 200 people/km^2^ were defined as urban, those with a population density higher than 10 people/km^2^ were defined as suburban, and those with a lower population density were defined as rural. Ulaanbaatar was defined as urban, Darkhan-Uul and Orkhon were defined as suburban, and other locations were defined as rural.

### Analysis

**Fig. 2:**
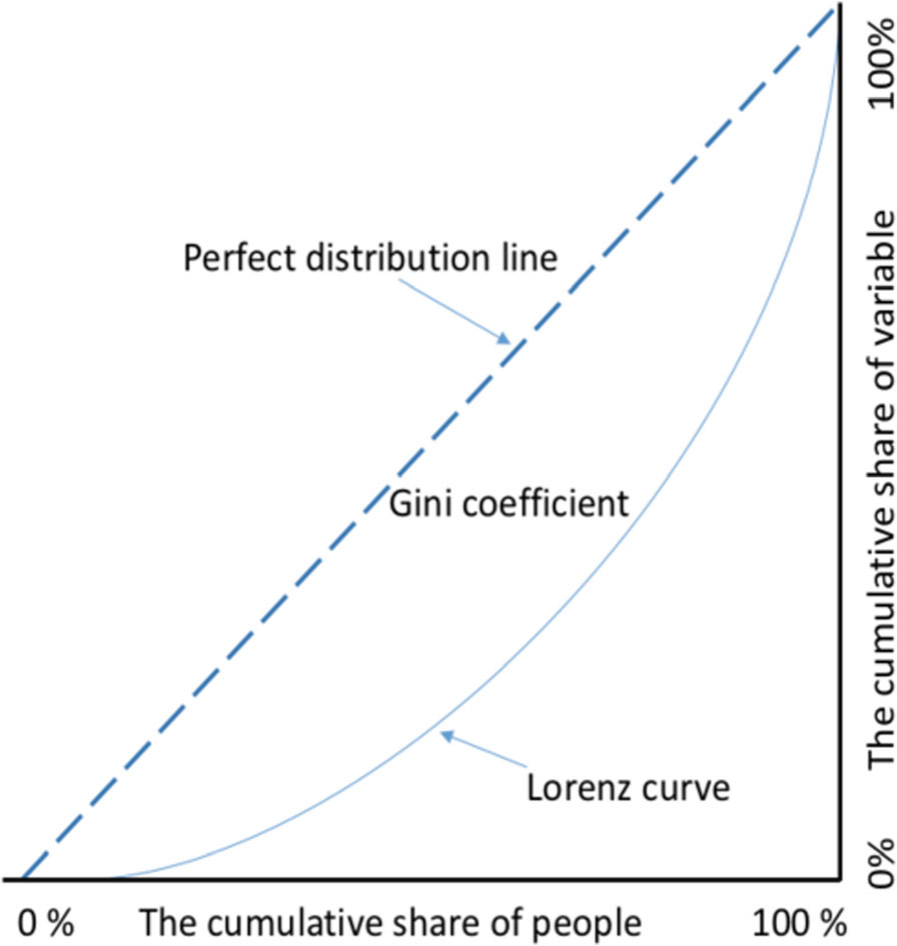
Lorenz curve and Gini coefficient. Source: Gini C, On the measurement of concentration and variability of characters (Translation by Giovanni Maria) [13]

First, the Mann–Whitney *U* test was employed to compare distributions between urban and rural areas. Then, distribution equality was determined using the Gini coefficient, one of the most common measures of distribution [4, 9–12], which was derived from the Lorenz curve . The Gini coefficient measures the area between the Lorenz curve and a hypothetical line of absolute equality, which is expressed as a percentage of the maximum area under the line. The Gini coefficient ranges from 0 to 1, with 0 representing perfect equality and 1 indicating perfect inequality [13]. In our study, the *x*-axis illustrates the cumulative share of the population and area of all of the provinces, and the *y*-axis illustrates the cumulative share of health care resources. Two indicators were calculated: the distribution of health resources per 10 000 population and the distribution of health resources per 1000 km^2^ area. Lorenz curves for each health care resource were created from the cumulative number of health care resources per population and area shares. The Gini coefficient was calculated using the following formula for each health care resource [13] (Fig. 2):

$$ R=\frac{\varSigma_{l=1}^S\left({i}_{l-1}+{i}_l-1\right){f}_l{x}_l}{\left(n-1\right){A}_n}-1 $$

We performed a cluster analysis to determine cut–off values, and differences among the provinces were depicted on a map using these cut–off values to show contrasts in distribution density.

## Results

The urban area in the study had the highest numbers of physicians (42.4) and hospital beds (77.6), but one rural province, namely Gobi-Altai, had the highest number of nurses (44.3) per 10 000 population, on average. Suburban areas had slightly higher numbers of these resources than did the rural provinces, with the exception of hospital beds. Rural areas had the lowest numbers of resources, on average.

In terms of the distribution of physicians and nurses per 1000 km^2^ area, the urban study area had 1228 and 1185, suburban areas had 193 and 260, and rural areas had 2.7 and 4, respectively. Further, the number of hospital beds was 2248 in the urban area, 453 in suburban areas, and 7.4 in rural areas, on average.

Results from the *U* test showed that, in terms of the distribution per population, there was a statistically significant difference only for physicians (*P* = 0.04); the distributions of nurses and hospital beds were not statistically different in urban and rural areas. In contrast, in terms of the distribution per area, there were statistically significant differences for all three health resources (*P* = 0.007) by location type.

Table 1 presents a comparison of health care resources in urban, suburban, and rural areas. The data are sorted from highest to lowest by the number of physicians per area.

**Table 1:** Comparison of health care resources and population size by province

Category	Province	Number of population	Area (km)	Demographic density/1 km	Total number of physicians	Number of physicians /10 000 pop	Number of physicians /1 000 km	Total number of nurses	Number of nurses /10 000 pop	Number of nurses /1 000 km	Total number of hospital beds	Number of hospital beds /10 000 pop	Number of hospital beds /1 000 km
Urban	Ulaanbaatar	1 362 974	4 704	306.5	5 779	42.4	1 228.5	5 575	40.9	1 185.1	10 577	77.6	2 248.4
Suburban	Orkon	94 421	840	127.2	260	27.5	309.1	342	36.2	406.9	592	62.7	704.8
	Darkhan-Uul	99 947	3 280	30.9	253	25.3	77.1	375	37.5	114.3	660	66	201.1
Mean in suburban		97 184	2 060	79.05	257	26.4	193.1	359	36.9	260.6	626	64.4	452.9
Rural	Gobisumber	16 058	5 540	3.1	60	37.6	10.9	68	42.1	12.2	89	55.7	16.1
	Selenge	106 212	41 200	2.6	192	18.1	4.7	286	26.9	6.9	630	59.3	15.3
	Uvurkhangai	112 992	62 900	1.8	229	20.3	3.6	329	29.1	5.2	720	63.7	11.4
	Bayan-Ulgii	95 151	45 700	2.2	158	16.6	3.5	287	30.2	6.3	671	70.5	14.7
	Arkhangai	93 086	55 300	1.7	167	17.9	3	281	30.2	5.1	532	57.2	9.6
	Tuv	90 107	74 000	1.2	188	20.9	2.5	302	33.5	4.1	533	59.2	7.2
	Khovd	81 479	76 900	1.1	181	22.2	2.4	280	34.4	3.6	574	70.5	7.5
	Bulgan	60 494	48 700	1.2	111	18.4	2.3	217	35.8	4.4	349	57.7	7.2
	Khuvsgul	126 043	100 600	1.3	223	18.4	2.2	359	28.5	3.6	691	54.8	6.9
	Uvs	75 792	69 600	1.2	145	18.4	2.1	274	36.2	3.9	521	68.8	7.5
	Khentii	71 212	80 300	0.9	155	18.4	1.9	242	34	3	416	58.4	5.2
	Zavkhan	69 732	82 500	0.9	159	18.4	1.9	270	38.7	3.3	592	84.9	7.2
	Dornogobi	63 808	109 500	0.6	198	18.4	1.8	200	31.3	1.8	401	62.8	3.7
	Dundgobi	44 351	74 700	0.6	121	18.4	1.6	164	37	2.2	247	55.6	3.3
	Sukhbaatar	57 423	82 300	0.7	126	18.4	1.5	211	36.7	2.6	379	66	4.6
	Bayankhongor	83 044	116 000	0.7	169	18.4	1.5	306	36.8	2.6	480	57.8	4.1
	Dornod	75 194	123 600	0.6	174	18.4	1.4	274	36.5	2.2	438	58.3	3.5
	Gobi-Altai	56 735	141 400	0.4	166	18.4	1.2	251	44.3	1.8	386	68.1	2.7
	Umnugobi	59 694	165 400	0.4	150	18.4	0.9	160	26.8	1	412	69.1	2.5
Mean in rural		75 716	81 902	1.2	162	18.4	2.7	251	34.2	4	477	69.9	7.4
*U* test result						^★^	^★★^			^★★^			^★★^
Total		2 995 949	1 564 964		9 364			11 053			20 890		

Mann–Whitney *U* test: urban + suburban vs. rural; ^★^ *P* < 0.05, ^★★^ *P* < 0.01

**Fig. 3:**
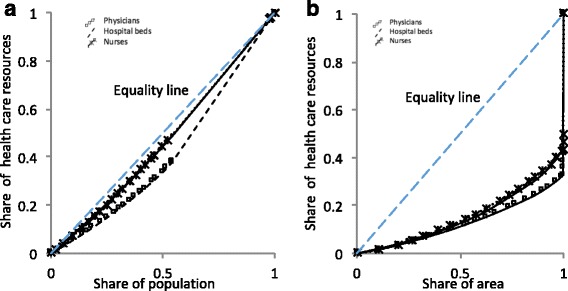
Lorenz curves for health care resource distribution per share of the population and area. Source: The Ministry of Health [6]. **a** (distributions per share of the population) and **b** (distributions per share of the area) illustrate the gap between the real and ideal distributions for the three health care resource variables

The Lorenz curves of the cumulative share of health care resources per population and area shares are shown in Fig. 3.

The Lorenz curves depicting health care resources per share of the population were close to the equality line for all three variables (Fig. 3a). This indicates that the three resources were equally distributed among the entire population. The distribution of physicians per share of the population had a Gini coefficient of 0.18, indicating equality. In contrast, the distribution of physicians per share of the area had a Gini coefficient of 0.74, indicating high inequality (Table 2).

**Table 2:** Equality of the distribution of health care resources by population and area

Indicators	Gini coefficient	
	Population	Area
Number of physicians	0.18	0.74
Number of nurses	0.07	0.67
Number of hospital beds	0.06	0.69

Unlike the curves for distribution by population share, the Lorenz curves showing health care resources per share of the area were far from the ideal line for all three variables; health care resources were found to be unequally distributed across geographical areas (Fig. 3b). The Gini coefficients for the distribution of nurses and hospital beds by share of the population were 0.07 and 0.06, respectively. In contrast, the Gini coefficients for the distribution of nurses and hospital beds by share of the area were 0.67 and 0.69, respectively.

**Fig. 4:**
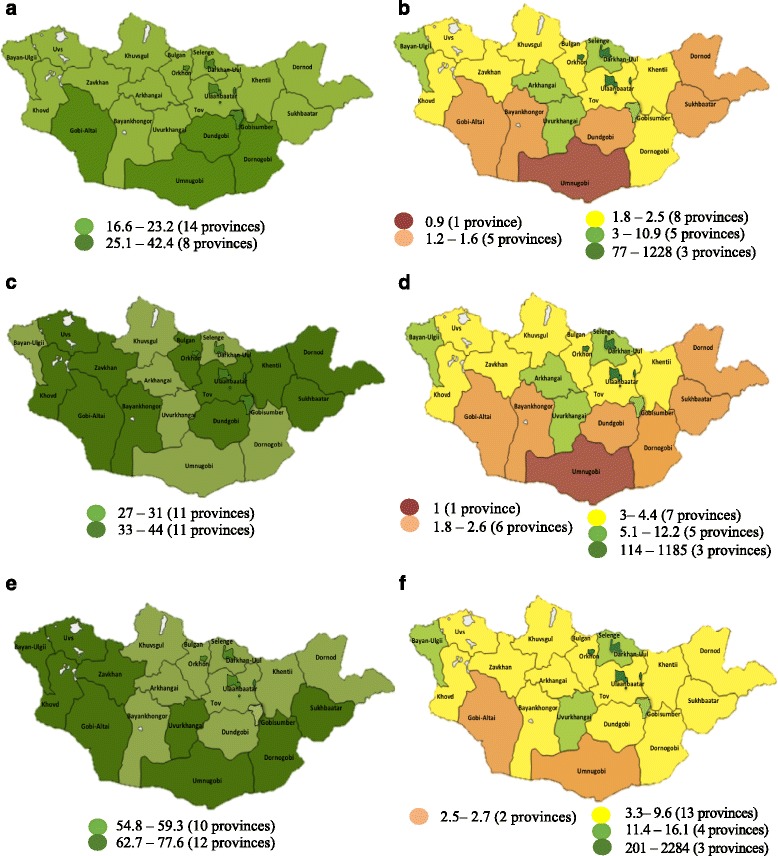
**a**–**f** Distribution of health care resource variables per share of the population and area. Source: The Ministry of Health [6] and National Statistical Information Service of Mongolia [5]. Distributions of the three health care resource variables are visualized on a map, using the cluster analysis to highlight gaps. Differences among the provinces are visualized using red and green colors based on the distribution density. Red represents an inadequate distribution (≤ 1 per area unit), and green represents an adequate distribution. Yellow and orange, as transition colors between green and red, represent an average health care resource supply

Comparing Fig. 4a and b shows that the distribution of physicians per 10 000 population (minimum = 16.6, maximum = 42.4) was found to be better balanced than was the distribution of physicians per 1000 km^2^ area (minimum = 0.9, maximum = 1228), which had a very large range, as is depicted using multiple colors on the map.

Similarly, comparing Fig. 4c and d shows that the distribution of nurses per 10 000 population (minimum = 27, maximum = 44) was more balanced than was the distribution of nurses per 1000 km^2^ area (minimum = 1, maximum = 1185), which was highly imbalanced, as is shown using multiple colors on the map.

Further, Fig. 4e illustrates that the distribution of hospital beds per 10 000 population (minimum = 54.8, maximum = 77.6) was nearly balanced, with only slight differences across the provinces. In contrast, the distribution per 1000 km^2^ area (minimum = 2.5, maximum = 2284) was highly imbalanced, as can be seen in Fig. 4f.

**Fig. 5:**
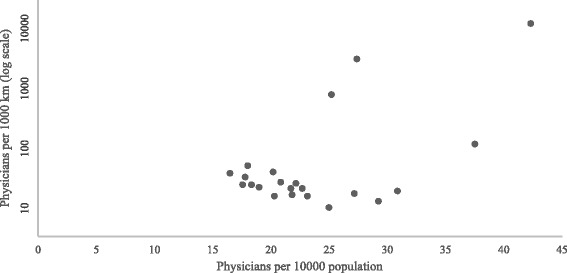
Distribution of physicians in Mongolia. Source: The Ministry of Health [6]. The distributions of physicians per population and per area units were plotted in Fig. 5, and the data were transformed using the logarithmic function

There was a statistically significant difference in the distribution of physicians in urban and rural areas, with urban areas having the highest number of physicians (Table 1, Fig. 5). The suburban areas also had higher numbers of physicians than did the rural provinces. Gobisumber was the rural area with the lowest number of physicians. However, the number of health resources per population unit was high in Gobisumber because of the province’s small population.

## Discussion

### Distribution of health care resources in Mongolia

Our results show that human resources exceeded the target numbers set in the Human Resource Policy of the Health Sector in Mongolia [14]. Additionally, the distributions of the three examined resources per 10 000 population were found to be equal throughout the country, based on the calculated Gini coefficients.

However, the geographical distributions of the three resources per 1000 km^2^ area were markedly different across the country. Geographical difficulties, extreme weather conditions (with temperatures as low at − 40 °C and as high at 35 °C), and limited transportation have created obstacles for the population in distant places in terms of accessing health services [12]. In addition, certain cultural and social factors [15, 16], especially the nomadic lifestyle, might account for the gap between rural and urban areas. An inherently nomadic lifestyle is a unique feature in rural areas, where herders are not rooted in a permanent setting across the seasons; rather, these people must move to a new place to provide food for their livestock and to maintain their livelihood. In this context, imbalanced distributions of health care resources per geographical area may be a barrier contributing to the disproportionate accessibility of health care services, especially in rural areas.

### International comparisons

The unideal allocation of health service providers at national level is a global, long-established, and grave problem. Regardless of how developed or rich countries are, higher proportions of health personnel are found in urban areas with better facilities [9, 17–19]. According to the World Health Organization’s (WHO) World Health Report [20], an estimate of 22.8 physicians, nurses, and midwives per 10 000 people is the minimum standard for achieving essential health interventions in those countries most in need. In a recent publication by the WHO [21], this minimum threshold for the health workforce requirements has been updated to 44.5 for achieving universal health coverage and reaching the Sustainable Development Goals 2016–2030. Mongolia was found to be close to the ideal point in terms of both numbers and adequate distribution for the three studied resources per population unit, with an average of 69.4 physicians and nurses per 10 000 people. Comparing our findings with those in a developed country, the Gini coefficient for physicians per population unit was 0.33 in Japan [11] and 0.18 in Mongolia.

We did not find any past work investigating the distribution of health care resources per area unit using the Gini coefficient. In the present study, we emphasized two types of distributions: per population unit and per area unit. Our results showed great differences between these two types of distributions. Further investigation is needed to determine the accessibility of health care resources and other contributing factors. This work could employ geographical information systems or other tools able to analyze both distribution and accessibility [22, 23]. Considering the nomadic lifestyle found among rural and remote populations in Mongolia may be critical for analyzing the distribution of health care resources.

### Recommendations

We recommend several provisions that interact with each other to achieve equality in the distribution of health care resources in Mongolia.

#### The mobile clinic

Mongolia can import accumulated practices from developed countries to increase the accessibility of health care services for its remote population; the mobile clinic would be the best method of health care provision in Mongolia. *Saiseimaru* is a mobile health ship that was equipped with professional teams to diagnose and cure conditions among people living on islands in Japan [24]. Adapted versions of *Saiseimaru*, such as a mobile bus or car, are needed in the Mongolian context, especially for herders living in remote sub-provinces and communes*.* The “Mobile Clinic” project [25] was initiated and implemented to fight adverse circumstances in six rural provinces of Mongolia. With this project, the Minister of Health approved a procedure that advanced the legal environment for delivering universal access to better quality health care services among the remote population. Based on these achievements, Mongolia must now move forward to the next step by implementing a nationwide, long-term program for delivering basic health care services throughout the year.

#### Rotational deployment procedures and higher incentives

Because all health care services are ultimately delivered by people, effective human resources management will play a vital role in the success of the health sector [3]. Regional disparities in the allocation of health resources might be a significant obstacle, preventing the rural population from accessing basic health care. Currently, “there is no efficient system for correcting the imbalance in the distribution of physicians in urban and rural areas” [11]. Mongolia has confronted this issue by devising rotational deployment procedures and a “calling service,” which allows physicians to be called from provincial hospitals or regional diagnostic and treatment centers to remote areas [26, 27]. However, the implementation and outcome of these procedures remain unclear. Stable rotational deployment procedures, where physicians—especially specialists—are dispatched from urban areas to treat the remote population within a certain time, are crucial. To achieve an equal distribution of existing human resources, collaboration with the Mongolian Health Workers’ Union, which acts for the protection of the rights, legal capacity, and social protection in labor relations of its members, is needed in the country.

Moreover, by definition, “rural and remote areas often convey a sense of isolation, both from a professional and personal point of view” [28]. On the professional level, career development, advancement opportunities, and the exchange of ideas with peers through networking have been considered of equal importance [9, 28, 29]. In Thailand, rural physicians have established their own society to support each other [29], and this society has been welcomed in public and medical arenas. Additionally, public recognition awards have been created, and some physicians have been recognized as the person of the year in Thailand at the national level. Thus, encouragement beyond salary, such as public recognition [30], flexible working hours [17], intensive training [28], additional cash bonuses [10, 29, 30] based on experience or length of commitment [17], and an adequate health infrastructure, are required [21, 26] in rural and remote areas. The Mongolian health sector currently has a few incentives: training, special awards, and cash bonuses [31–33]. However, additional incentives are needed for health workers in remote areas. Thus, the government of Mongolia should develop these kinds of encouraging incentives to reduce the geographical disparity and attract more medical personnel to rural areas.

## Conclusions

Although the distributions of health care resources per population were adequate for the population size, a striking difference was found in terms of the distributions per area. Because of the nomadic lifestyle among rural and remote populations in Mongolia, geographical imbalances need to be taken into consideration when formulating policy, rather than simply increasing the number of health care resources.

Studies such as ours can be used as a basis for health systems planning to correct the unequal distribution of health care resources. Additional studies should be done continuously and should incorporate other types of health care resources, including technological resources and financing, to identify the overall circumstances of health resources in the country.

## Abbreviation

WHO: World Health Organization
